# Still slow, but even steadier: an update on the evolution of turtle cranial disparity interpolating shapes along branches

**DOI:** 10.1098/rsos.170899

**Published:** 2017-11-29

**Authors:** Christian Foth, Eduardo Ascarrunz, Walter G. Joyce

**Affiliations:** 1 Departement für Geowissenschaften, Universität Freiburg, 1700 Freiburg, Switzerland; 2 Staatliches Museum für Naturkunde, Rosenstein 1, D-70191 Stuttgart, Germany

**Keywords:** turtle skulls, geometric, morphometrics, disparity through time, branch interpolation

## Abstract

In a previous study, we estimated the cranial disparity of turtles (Testudinata) through time using geometric morphometric data from both terminal taxa and hypothetical ancestors to compensate for temporal gaps in the fossil record. While this method yielded reasonable results for the Mesozoic and the early Cenozoic, we found a large drop in cranial disparity for the Miocene, for which we found no correlation with known environmental changes or extinction events. Instead, we speculated that the Miocene dip was a result of poor sampling of fossils or ancestors in this time bin. To countervail this problem, we here updated our original dataset and interpolated changes of shape along the branch lengths and compared them with the previous data. We furthermore explored the impact of topological and temporal uncertainty, demonstrating that the Miocene dip, indeed, is a sampling artefact. All remaining conclusions of the previous study could be more or less supported, nevertheless, including an apparent correlation with global biogeographic events, a minor correlation between cranial disparity and global temperature, and resilience across the K/T extinction event.

## 1. Introduction

**Figure 1.**
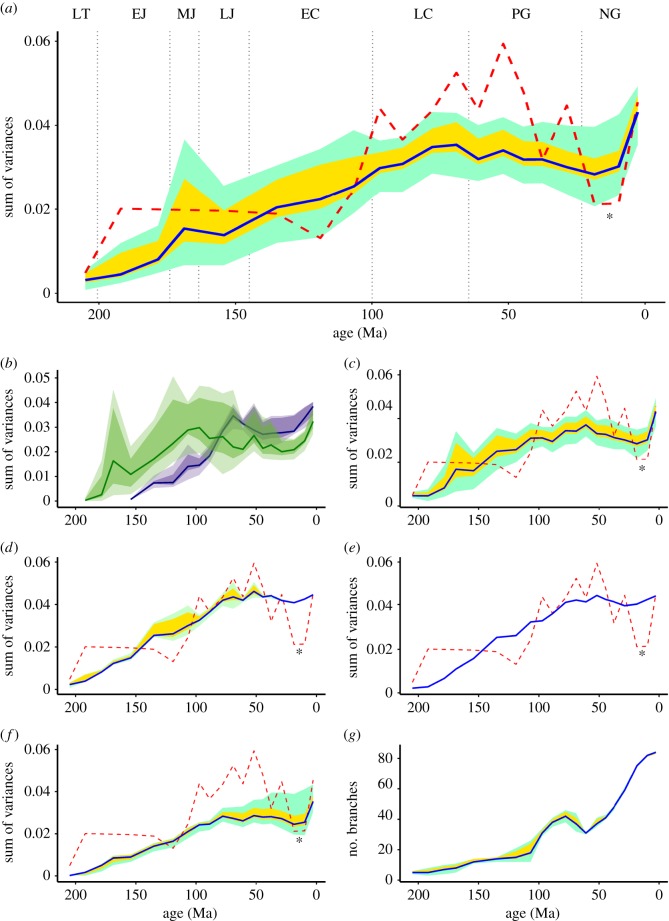
(*Caption overleaf.*)

Over the course of the last decades, the combination of geometric morphometrics with phylogenetic comparative methods has become a promising resource for the study of macroevolutionary dynamics, including the evolution of disparity, which quantifies morphological diversity as opposed to taxonomic, functional or phylogenetic diversity [1–5]. In comparison with studies of taxonomic diversity, however, studies of morphological disparity have only recently begun to make fuller use of a phylogenetic framework by including information from ghost lineages. Two of us (C.F. and W.G.J.) recently surveyed temporal changes to the cranial disparity of turtles using a sample of 172 fossil and recent species ranging from the Late Permian to the Recent [6]. As most clades were only sampled in a subset of time bins in which they must have occurred, we partially accounted for ghost lineages by populating certain time bins with the hypothetical ancestors they contained using current phylogenetic hypotheses. In the resulting disparity curves, we identified three evolutionary phases for the turtle cranium: a gradual increase of cranial disparity from the Late Triassic to the Palaeogene with only a minor perturbation at the K/T extinction event, a strong decrease in cranial disparity from the Eocene to the Miocene, and a strong recovery towards the Recent (figure 1*a*). Given that a correlation with climate was not strongly supported, we suggested instead that cranial disparity was perhaps driven by biogeography, as changes in disparity correlate well with the biogeographically controlled origin and extinction of turtle lineages [8]. We also disregarded the dip in cranial disparity in the Miocene as an artefact of the methods we used, as fossils and ancestors were only poorly sampled in the Miocene time bin. However, if the Miocene can be disregarded due to sampling biases, how much confidence should be accorded to the other time bins?

**Figure 1.** (*Overleaf.*) Temporal disparity of turtle skull shape in lateral view, as measured from phylogenetically interpolated values (‘gradualistic’ model), with disparity quantified as the sum of variance of shape variables (*a*–*f*). The blue solid line shows the median estimated sum of variances of the bin, with the yellow ribbon bands showing the total range of the sum of variances estimated from 1000 trees (see below), and the cyan colour bands showing the range delimited by the 2.5% and 97.5% quantiles of the same. The red dashed lines show the sum of variances previously estimated in Foth and Joyce [6] with the Miocene dip (asterisk). (*a*) Sum of variances for all representatives of turtles (Testudinata), computed from a set of trees that vary in their random resolution of polytomies, fossil ages randomly drawn from their full range of possible time of appearance, and minimum node age constraints drawn from the molecular clock analysis of Pereira *et al.* [7] (tree set #3). (*b*) Sum of variances for Pan-Cryptodira (green) and Pan-Pleurodira (purple) using the same trees. (*c*) Skull shape disparity of Testudinata computed from trees as in (*a*), but with fossil ages set to their earliest possible values (tree set #1). (*d*) Skull shape disparity of Testudinata computed from trees as in (*a*), but with polytomies kept unresolved (tree set #2). (*e*) Skull shape disparity of Testudinata computed from a single time-scaled supertree with unresolved polytomies. (*f*) Lateral skull shape disparity of Testudinata computed from trees as in (*a*), but excluding fossils. (*g*) Number of samples per bin (i.e. number of branches intersecting the midpoint of the bin) computed from the same trees as in (*a*) (tree set #3). Meaning of the ribbon bands analogous to the above. LT, Late Triassic; EJ, Early Jurassic; MJ, Middle Jurassic; LJ, Late Jurassic; EC, Early Cretaceous; LC, Late Cretaceous; PG, Paleogene; NG, Neogene.

Two recent developments provide an incentive to revisit our original dataset. First, Wilberg [9] presented a new method based on Friedman [10] that fully embraces phylogenetic data by interpolating traits between nodes along the branches. And second, Pereira *et al.* [7] presented a molecular calibration analysis that estimates the divergence dates of nearly all species of living turtles. As both factors allow addressing issues we noted before, we here present a brief follow-up analysis. Finally, we explore possible correlation of cranial disparity in turtles with climate and biogeography.

## 2. Material and methods

The materials and methods in general follow Foth and Joyce [6] (see electronic supplementary material, S1–S4 of [6]) with the exception of the following modifications. We first restricted our analysis to the lateral view of the skull, as our initial analysis concluded this view to have the highest disparity and because the three skull views included in the initial dataset revealed similar trends [6]. We furthermore restricted our analysis by excluding *Eunotosaurus africanus* (Late Permian) and *Pappochelys rosinae* (Middle Triassic) as both taxa fall far outside the time binning scheme relevant to turtles (Testudinata). As the full utilization of ghost lineages is expected to have an impact on deep divergences, we furthermore adjusted the phylogenetic position of some extinct marine turtles, in particular by resolving pan-chelonioids following Weems & Brown [11] and by placing protostegid turtles, including Mesozoic ‘dermochelyoids’ [12,13], as sister to thalassochelydians, as originally proposed a decade ago [14]. We finally time-scaled our supertree using the bin_timePaleoPhy function of the package paleotree v. 2.7 [15] in R [16]. In addition to using stratigraphic ages (midpoint of occurrence) of terminal taxa (with ages of extant turtles set to zero) taken from the literature (see electronic supplementary material S1 of [6]), the time-scaling of the tree was undertaken by enforcing node age estimates from a sample of 1000 post-burn-in MCMC trees from the molecular clock analysis of Pereira *et al.* [7], while the remaining nodes were calibrated by evenly distributing the available time between branches (the ‘equal’ time-scaling method implemented in bin_timePaleoPhy, with the ‘vartime’ parameter set to 1). In order to take into account uncertainties introduced by unresolved phylogenetic relationships in the supertree and inaccuracies associated with the age of fossils, we produced the following sets of time-scaled trees: (i) 1000 trees in which the polytomies of the supertree were resolved randomly prior to time-scaling, and the fossil observations were considered to have exactly the age of the lower boundary of the stratigraphic unit in which they occurred; (ii) 1000 trees in which the supertree polytomies were kept unresolved for time-scaling, and the ages of the fossil observations were drawn randomly from a uniform distribution spanning the duration of the stratigraphic unit in which the fossils were found; and (iii) 1000 trees in which polytomies were resolved as in (i) and fossil ages treated as in (ii).

As in our previous analyses, the first seven principal components containing significant shape information for the lateral view were used for the disparity analyses, which were selected on the basis of the broken-stick method [17] performed in PAST 3.05 [18]. The PC values were mapped onto the supertree using the anc.recon function from the new R package Rphylopars v. 0.2.9 [19], which computes maximum-likelihood (ML) estimates of nodal trait values assuming a Brownian motion model with a constant rate of diffusion. Under this condition, ML is equivalent to squared-change parsimony [10,20] which was used in the previous study [6]. Using a linear interpolation on the time-scaled tree branches (‘gradual model’ of Friedman [10]), the PC values observed and reconstructed at the nodes of the tree were then used to compute the principal components along the branches of the tree, in particular at the midpoint of each time bin crossed by a particular branch [9,10]. Because the phylogeny is characterized by many long ghost lineages, the ‘punctuated’ model was not applied as it would imply unrealistically extended evolutionary stages for the turtle cranium. The sum of variance was calculated for each bin using the branch interpolation values at the midpoint of the temporal duration of the bin to trace disparity through time as this disparity metric is relatively robust to uneven spatio-temporal sampling of the fossil record [21]. In this regard, we sample differently relative to Foth and Joyce [6] by omitting terminals and nodes from the computation of the sum of variances.

As in the previous version, we tested for a correlation between cranial disparity and climate using *δ*^18^O records of Veizer *et al.* [22] and Zachos *et al.* [23] as proxies for global temperature. In addition, we further tested for the relationship between cranial disparity and the number of major, unconnected continental landmasses populated by turtles per time bin, using current palaeogeographic reconstructions [24–26]. In both cases, the datasets were compared with the help of Spearman's rank-order correlation test using PAST and ordinary and generalized least square regression (OLS and GLS) analyses using the nlme package v. 3.1-126 [27] for R (see electronic supplementary material S1, Text S1 for more details).

## 3. Results

Unless otherwise stated, the presentation of our main results is drawn from the analysis of the set of time-scaled trees #3 with the ‘gradualistic’ model, therefore taking into account the effect of different possible topologies and ages for fossils and internal nodes. The median curve shows a relatively steady increase of disparity in the skulls of turtles from the Late Triassic to the Maastrichtian (latest Cretaceous) with minor fluctuations (figure 1*a*, solid blue line). Thus, the new median curve differs from the original one by lacking a period of stagnation from the Early Jurassic to the Early Cretaceous (figure 1*a*, dashed red line). As in the original analyses, only a minor (possibly artefactual) perturbation is apparent at the K/T extinction event, but the peak of cranial disparity is much lessened, and shifted from early Eocene to the Maastrichtian (latest Cretaceous). In contrast to the original analyses, the median curve is characterized by stagnation during the Cenozoic at a level slightly below that of the Maastrichtian (latest Cretaceous), but above that of all Early Cretaceous time bins. However, a minor drop, much less dramatic than in the original study, is still present during the Oligocene and Miocene, but still at the disparity level of the Cenomanian (Late Cretaceous). After this minor dip, cranial disparity recovers completely and even surpasses all previous values (see electronic supplementary material S1, table S2). The full set of curves drawn from the 1000 trees spans wide ranges of values of sum of variance through all the bins, indicating a high degree of uncertainty in the trajectory of disparity over time, and therefore precluding the attribution of much biological significance to the minor fluctuations observed.

When phylogenetic and temporal uncertainty is omitted from consideration (figure 1*e*, curve computed from the base supertree), the curve shows the same general trends, but disparity is overall higher and extant disparity equals that of the Eocene maximum. The effect of uncertainty to the age of fossils is minor and more pronounced in the time bins that include many fossils (figure 1*d*, curve computed from the set of time-scaled trees #2). The effect of phylogenetic uncertainty, on the other hand, is drastic (figure 1*c*, curve computed from the set of time-scaled trees #1), representing the main source of observed uncertainty in our estimates of disparity. If disparity is estimated excluding fossils from the data, the overall shape of the curve is preserved, with fluctuations further smoothed down (figure 1*f*). This indicates that the recovered overall disparity pattern is dominated by the observations and relationships of the extant species, whereas the fossils seem to have a stronger influence on the uncertainty of the disparity estimates, which is augmented by the uncertainty in the topology. In all cases, the curves are much smoother than the original graph produced by Foth and Joyce [6], as should be expected from the use of interpolated ancestors.

As in the original analyses, the cranial disparity of pan-cryptodires exceeds that of pan-pleurodires over most of the time (figure 1*b*, solid lines). Pan-cryptodires show a steady increase in cranial disparity throughout the Cretaceous until achieving their maximum around the border of the Early and Late Cretaceous. Although a number of ups and downs are apparent throughout the Cenozoic, pan-cryptodires overall show a moderate decline until the Oligocene, before their disparity increase towards the Recent, again. Pan-pleurodires also show a steady increase throughout the Cretaceous and reach their first maximum during the Late Cretaceous. In contrast to the initial analysis, this clade seems not to be affected by the K/T extinction. The cranial disparity of the group declines during the Eocene, but recovers in the Recent time bin, surpassing the level of the Maastrichtian. The cranial disparity of pan-pleurodires surpasses that of pan-cryptodires since the Late Cretaceous (see electronic supplementary material S1, Table S1).

Like in the previous study, the relationship between skull shape disparity and climate is rather weak. In contrast to generalized least square regression, only OLS regression and Spearman's rank-order correlation test found weak correlations between the cranial disparity curves of Pan-Testudines (*R*^2 ^= 0.285; *p *> 0.015/*r_S_* = 0.466; *p *> 0.040) and pan-pleurodires (*R*^2 ^= 0.353; *p *> 0.015/*r_S_* = 0.547; *p *> 0.031) and climate data (see electronic supplementary material S1, Table S4, S5), while all GLS regression tests indicate no correlation. In addition, a specific comparison between changes in temperature and cranial disparity across successive time bins found no relationship, too. This is different for the comparison between cranial disparity and number of major landmasses through time, as both parameters are significantly correlated with each other on the basis of the OLS regression (*R*^2 ^= 0.616; *p *> 0.005) and Spearman's rank-order correlation test (*r_S_* = 0.715; *p *> 0.005) (see electronic supplementary material S1, table S6).

## 4. Discussion and conclusion

### 4.1. Comparisons with previous analysis

Although our new analysis broadly recovers results similar to those of our initial analysis, some notable differences are apparent that confirm that a fuller use of phylogenetic data has a broad impact on disparity analyses. As in our initial study, turtles show a slow but steady increase in cranial disparity throughout the Mesozoic, which opposes the near explosive achievement of high disparity levels in other groups of animals [28,29]. Such delayed peak of disparity was interpreted to indicate a concordance between morphological and taxonomic diversification (which shows an exponential-like shape through time, figure 1*g*), and thus implies neither constraints on morphological evolution nor trends in morphological step size during the prolonged period of increase [30]. In contrast to our initial analysis, however, our revised analysis reveals that turtles maintained a high level of disparity in skull shape throughout the Cenozoic, instead of showing a strong decline towards the Miocene, followed by a recovery towards the Recent. This result confirms our previous suspicion that our initial Cenozoic curve was negatively affected by poor sampling and that this sampling bias might be overcome by including ghost lineages. We initially hypothesized that the decrease of cranial disparity throughout the Cenozoic may have been caused by the loss of morphospace that occurred through the extinction of more basal groups (e.g. adocids and paracryptodires), but the newly recovered plateau during this time interval reveals that evolution in extant turtle clades seems to be able to compensate this loss.

Based on the simulations undertaken by Foote [31], the overall course of the disparity curve indicates that during the Mesozoic the morphological step size was relatively high, but constant with no temporal changes (see above). In contrast, the more or less constant level of cranial disparity during the Cenozoic indicates that the number of morphological steps was significantly reduced compared with the Mesozoic. As this pattern is also evident for the two subclades, it is evident that pan-cryptodires and pan-pleurodires underwent similar evolutionary patterns.

As we thought a climatological control of disparity to be biologically implausible, we hypothesized following our initial analysis [6] that cranial disparity may be correlated with biogeography. In particular, as most turtle groups populate different parts of the available morphospace [32], the increasing fragmentation of Pangaea over the course of the late Mesozoic may had led to the formation of increasing amounts of endemism. This trend was only reversed during the Cenozoic when the extinction of basally branching turtle groups, perhaps caused by the global spread of cryptodires made possible by emerging continental bridges, offset gains in cranial disparity [7,8]. This hypothesis can now be supported by a correlation between cranial disparity and number of major landmasses through time, although we lack an understanding of the underlying biological processes. Interestingly, the revised analysis still shows an overall poor correlation with temperature, thereby indicating once again that climate does not appear to directly control the disparity of turtle skulls. However, this does not necessarily mean that the general morphological diversity of turtles was completely unaffected by global temperature, especially as their geographical dispersal seems to show such a correlation [33]. Thus, to fully embrace how turtle anatomy may be affected by climate change over time, further studies are necessary focusing on different body parts, including the shell and limbs.

Although the use of ancestral lineages resulted in disparity curves that are much more gradual than ones we initially retrieved, some notable steps still remain, particularly in the curves of the two primary clades of extant turtles. Here, the skull shape disparity of pan-pleurodires seems to suffer a loss during the Palaeogene, probably due to the loss of marine-adapted lineages at that time (e.g. bothremydids, stereogenyines, etc.), but recovered fast due to the diversification of chelids. Interestingly, although pleurodires represent only about a quarter of all extant turtles [34], their sum of variance, a disparity measure that takes diversity into account, is greater than that of cryptodires for the whole Cenozoic.

### 4.2. Limitation of results

We noted following our initial analysis [6] that the outcome strongly depends on phylogenetic relationships, the algorithm of time-scaling [35] and, potentially, the method of ancestral reconstruction [36,37]. This is even more true for the current analysis, where we measured disparity exclusively from phylogenetically interpolated shape variables. Consequently, the new curves are characterized by much smoother trajectories than the previous one (figure 1*a*). Furthermore, the resolution of the polytomies in the supertree has a significant effect on the sum of variances measured from interpolated traits (figure 1).

Despite these uncertainties, we are confident regarding the robustness of our results for the following reasons. This and the previous analysis found disparity curves showing overall similar trends that are apparently independent from the particular topology use (see Material and methods), the ages of fossils, the use of branch interpolations or the addressing of topological uncertainty. The only major difference is the presence of a Cenozoic plateau in the present analysis. This confirms that the Miocene dip found in the original analysis indeed is a sampling artefact that can be addressed through the usage of branch interpolations.

We nevertheless see room for improvement, especially in regard to sampling. Whereas our study tightly samples the available morphospace among living turtles by including a representative of every ‘genus’, the vast majority of fossil turtle lineages remain poorly sampled, because skulls are either not known or too poorly preserved to allow inclusion. This is not a trivial concern, considering the often-bizarre morphology of numerous fossil turtle skulls not included in our study because of poor preservation (e.g. the pig-snouted *Nanhsiungchelys wuchingensis* [38]) or that fossil turtles only recently described still yield highly surprising morphologies (e.g. the crocodile-like *Ocepechelon bouyai* [12] or the broad-snouted *Alienochelys selloumi* [13]). While this sampling bias is inherent to any study of morphological or taxonomic diversity, we here identify another bias that may be overcome partially using phylogenetic data. As implemented herein, phylogenetic data allow sampling time bins not represented by fossil by including ghost lineages. However, we note that many time bins still remain unsampled, because the fossil sampled is not necessarily the last representative of its lineage. For example, we compensate for the absence of adocids in the majority of Cretaceous time bins, as the sole representative of the group in our sample, *Adocus lineatus*, only samples the latest Cretaceous time bin (Maastrichtian). However, the adocid lineage actually persists into the Eocene of Asia [39] and the unusual morphology of this lineage is therefore not accounted for in the Palaeogene time bins. Similarly, the solemydid lineage is known to persist to the Late Cretaceous (Maastrichtian) [40], but is last sampled herein in the Early Cretaceous (Aptian/Albian), the macrobaenid lineage is known to persist to the Paleocene [41], but is last sampled in the Late Cretaceous (Campanian), and the *Kallokibotion* lineage is thought to persist to the Paleocene [42], but is here last documented from the Late Cretaceous (Maastrichtian).

In summary, the interpolation of traits along branches is a useful method to minimize the effect of artefacts related to sampling gaps in the fossil record. Following Wilberg [9], it should be noted that reconstructed ancestors and interpolated traits are not meant to represent true ancestral shapes, but placeholders in the absence of sampled specimens. Indeed, the extensive use of phylogenetic interpolation implies that the results can be highly sensitive to the method of ancestral trait reconstruction. Regardless, in our appreciation, the use of such estimated morphologies for a given time bin is better than treating an absence of sampled data as actual absence of shape. Along those lines, the Miocene dip in the cranial disparity curves of turtles we retrieved in our original study [6] turns out to be a gap in the fossil record rather than a natural event. Our updated disparity curve otherwise broadly supports the trends we observed during our initial study, including the slow and steady increase of cranial disparity throughout the whole Mesozoic, the negligible impact of the K/T extinction event and a weak correlation with global temperature. As a consequence, the new analysis is still compatible with our original hypothesis that the cranial disparity of turtles could be driven by biogeographic factors, while climate played only a secondary role.

## Supplementary Material

Foth-et-al-Turtle-Skull-disparity-S1

## Data accessibility

Additional results supporting this article have been uploaded as part of the electronic supplementary material. Original data are available at the electronic supplementary material of Foth and Joyce [6], while R scripts and all relevant input and output files can be found in the following repository: https://bitbucket.org/eascarrunz/fothetal2017_turtle_disparity.
