# Supplementary material for: Still slow, but even steadier: an update on the evolution of turtle cranial disparity interpolating shapes along branches
Source: R Soc Open Sci. 2017 Nov 29;4(11):170899. doi: 10.1098/rsos.170899 (PMC5717657; doi:10.1098/rsos.170899)
Supplement: Foth-et-al-Turtle-Skull-disparity-S1 [file rsos170899supp1.pdf]

Electronic Supplement Material S1

Still slow, but even steadier: An update on the evolution of turtle cranial disparity interpolating shapes along branches

Christian Foth, Eduardo Ascarrunz, Walter G. Joyce

Content

Table S1 Binning of geological ages
Table S2 Temporal disparity (sum of variances) of turtle skulls in lateral view
Text S1 The impact of sample size, climate and biogeogrpahy on cranial shape disparity
Table S3 Correlation between skull shape disparity changes and sample size
Table S4 Correlation between skull shape disparity and $\delta^{18}O$
Table S5 Correlation between skull shape disparity changes and $\delta^{18}O$ changes
Table S6 Correlation between skull shape disparity changes and number of major landmasses through time
References

Table S1. Binning of geological ages. *For the Late Triassic all estimated traits of hypothetical ancestors and branches were sampled at the midpoint of the Rhaetian, while the only terminal taxa *Proganochelys quenstedti* extisted in the late Norian.

Bin	Ages	Mid-age
Recent	QUATERNARY–PLIOCENE	2.7
Miocene-I	MESSINIAN–SERRAVALLIAN	9.6
Miocene-II	LANGHIAN–AQUITANIAN	18.4
Oligocene	CHATTIAN–RUPELIAN	28.5
Eocene-I	PRIABONIAN–BARTONIAN	37.6
Eocene-II	LUTETIAN	44.5
Eocene-III	YPRESIAN	51.9
Palaeocene	THANETIAN–DANIAN	61.0
Late Cretaceous-I	MAASTRICHTIAN	69.1
Late Cretaceous-II	CAMPANIAN	77.9
Late Cretaceous-III	SANTONIAN–TURONIAN	88.8
Late Cretaceous-IV	CENOMANIAN	97.0
Early Cretaceous-I	ALBIAN	106.5
Early Cretaceous-II	APTIAN	119.5
Early Cretaceous-III	BARREMIAN–BERRIASIAN	135.5
Late Jurassic	TITHONIAN–OXFORDIAN	154.5
Middle Jurassic	CALLOVIAN–AALENIAN	169.0
Early Jurassic-I	TOARCIAN	178.5
Early Jurassic-II	PLIENSBACHIAN–HETTANGIAN	192.0
Late Triassic	RHAETIAN–NORIAN*	205.0

Table S2. Temporal disparity (median of sum of variances and differences of sum of variances) of the skull (lateral view) of Testudinata, Pan-Pleurodires and Pan-Cryptodires.

	**Testudinata**		**Pan-Pleurodires**		**Pan-Cryptodires**	
**Bin**	**SumV**	**ΔSumV**	**SumV**	**ΔSumV**	**SumV**	**ΔSumV**
Recent	0.043	-0.013	0.038	-0.005	0.032	-0.008
Miocene-I	0.030	-0.002	0.034	-0.005	0.024	-0.004
Miocene-II	0.028	0.002	0.028	-0.001	0.021	-0.001
Oligocene	0.030	0.002	0.028	0.000	0.020	0.003
Eocene-I	0.032	0.000	0.027	0.000	0.023	-0.001
Eocene-II	0.032	0.002	0.027	0.002	0.021	0.005
Eocene-III	0.034	-0.002	0.029	0.003	0.027	-0.006
Paleocene	0.032	0.003	0.031	0.003	0.021	0.001
Late Cretaceous-I	0.035	-0.001	0.035	-0.006	0.022	0.004
Late Cretaceous-II	0.035	-0.004	0.029	-0.010	0.026	-0.001
Late Cretaceous-III	0.031	-0.001	0.018	-0.004	0.025	0.004
Late Cretaceous-IV	0.030	-0.004	0.015	-0.001	0.030	-0.001
Early Cretaceous-I	0.025	-0.003	0.014	-0.007	0.029	-0.005
Early Cretaceous-II	0.022	-0.002	0.008	0.000	0.023	-0.006
Early Cretaceous-III	0.020	-0.007	0.007	-0.007	0.017	-0.006
Late Jurassic	0.014	0.002	0.001	NA	0.011	0.005
Middle Jurassic	0.015	-0.007	NA	NA	0.016	-0.014
Early Jurassic-I	0.008	-0.004	NA	NA	0.003	-0.002
Early Jurassic-II	0.004	-0.001	NA	NA	0.000	NA
Early Triassic	0.003	NA	NA	NA	NA	NA

Text S1. The impact of sample size, climate and biogeogrpahy on cranial shape disparity

To evalutate how the evolutionary trends of cranial shape disparity are affected we tested the correlation between sample size (Table S3), climate change (Table S4, S5) and biogeogrpahy (Table S6). To test the impact of climate change on cranial disparity, we used the geochronological $\delta^{18}O$ data based on an updated dataset of Veizer et al. (2000) (http://mysite.science.uottawa.ca/jveizer/isotope_data/), ranging from the Late Triassic to Recent, and the original data set of Zachos et al. (2001) for the Cenozoic. $\delta^{18}O$ data were averaged according to the binning scheme of the disparity analysis (see Table S1). For the biogeographical comparison we used the number of independent landmasses for each bin based on Briggs (1995), Ali & Aitchison (2008) and Mosar et al. (2002).

Correlations were tested between absolute values (all comparisons) and differences between subsequent time bins (for comparison with climate change and biogeography) using Spearman's rank-order correlation test, ordinary least squares (OLS), generalized least squares (GLS) regression analyses with a first-order autoregressive model. Spearman's rank-order correlation test is a linear correlation test based on ranks, in which the r_s coefficient describes the strength of the correlation. The test ranges from -1 to 1, in which $r_s = 0$ indicates no correlation (Hammer & Harper 2006). The test was excecuted in *PAST* (Hammer et al. 2001). In contrast, OLS estimates the strength of serial correlation between two parameters by minimizing the sum of squares from a 'random' error (Hammer & Harper 2006), while GLS uses maximum likelihood to correct for non-independence between adjacent points (Hansen 2007). The strength of correlation is given in form R^2 for OLS and φ (dispersion parameter) and a correlation parameter for GLS. In addition, *p* values report, how significantly different the estimated regession slopes are from zero. All regression analyses were performed in *R* (R Development Core Team 2011), in which GLS was performed using the *gls* function of the package '*nlme*' (Pinheiro et al. 2016).

Table S3. Results of ordinary least squares (OLS) and generalised least squares (GLS) regression, and Spearman's correlation of skull shape disparity (based on median of sum of variances) and sample size (based on number of branches) per bin. Bold values represent significant changes.

	OLS			GLS				Spearman's	
	Slope	*p* value	R^2	Slope	*p* value	PHI	Correlation	r_S	*p* value
**Branches**	**1732.81**	**0.000**	**0.576**	345.47	0.342	1.000	0.000	**0.769**	**0.000**

Table S4. Results of ordinary least squares (OLS) and generalised least squares (GLS) regression, and Spearman's correlation of skull shape disparity (based on median of sum of variances) on the $\delta^{18}O$ palaeotemperature proxy based on Veizer et al. (2000) and Zachos et al. (2001). Bold values represent significant changes.

		OLS			GLS				Spearman's	
	SumV vs. $\delta^{18}O$	Slope	*p* value	R^2	Slope	*p* value	PHI	Correlation	r_S	*p* value
**Veizer**	**Pan-Testudines**	**78.60**	**0.015**	**0.285**	40.19	0.304	1.000	0.000	**0.466**	**0.040**
	**Pan-Pleurodires**	**87.30**	**0.015**	**0.354**	72.30	0.075	0.984	-0.204	**0.547**	**0.031**
	**Pan-Cryptodires**	52.71	0.247	0.078	-1.04	0.976	1.000	0.000	0.046	0.854
**Zachos**	**Pan-Testudines**	91.77	0.356	0.142	47.60	0.394	1.000	0.000	-0.214	0.619
	**Pan-Pleurodires**	183.11	0.077	0.430	143.64	0.110	1.000	0.000	0.405	0.327
	**Pan-Cryptodires**	115.84	0.288	0.185	11.182	0.865	1.000	0.000	0.119	0.793

Table S5. Results of ordinary least squares (OLS) and generalised least squares (GLS) regression, and Spearman's correlation of skull shape disparity changes (based on median of sum of variances) on the $\delta^{18}O$ changes between subsequent time bins based on Veizer et al. (2000) and Zachos et al. (2001). Bold values represent significant changes.

		OLS			GLS				Spearman's	
	**ΔSumV vs. $\Delta\delta^{18}O$**	**Slope**	***p* value**	R^2	**Slope**	***p* value**	**PHI**	**Correlation**	r_S	***p* value**
**Veizer**	**Pan-Testudines**	9.97	0.815	0.003	13.45	0.748	0.206	-0.423	-0.026	0.917
	**Pan-Pleurodires**	-53.37	0.272	0.092	-88-913	0.057	0.531	0.363	-229	0.411
	**Pan-Cryptodires**	19.11	0.647	0.013	13.59	0.738	0.244	0.180	0.078	0.757
**Zachos**	**Pan-Testudines**	-9.18	0.954	0.001	-1.58	0.993	0.126	-0.998	-0.464	0.302
	**Pan-Pleurodires**	118.98	0.638	0.289	118.98	0.638	0.000	-0.996	0.500	1.000
	**Pan-Cryptodires**	132.77	0.253	0.250	132.42	0.257	-0.025	-0.996	0.143	0.783

Table S6. Results of ordinary least squares (OLS) and generalised least squares (GLS) regression, and Spearman's correlation of skull shape disparity (based on median of sum of variances) on the number of landmasses (N) based on Ali & Aitchison (2008). Bold values represent significant changes.

	OLS			GLS				Spearman's	
	**Slope**	***p* value**	R^2	**Slope**	***p* value**	**PHI**	**Correlation**	r_S	***p* value**
**SumV vs. N**	**141.20**	**0.000**	**0.616**	43.85	0.332	0.973	-0.299	**0.715**	**0.000**
**ΔSumV vs. ΔN**	40.72	0.453	0.033	30.60	0.548	0.344	-0.376	0.340	0.15

References

Ali, J. R. and Aitchison, J. C. 2008 Gondwana to Asia: plate tectonics, paleogeography and the biological connectivity of the Indian sub-continent from the Middle Jurassic through latest Eocene (166–35 Ma). *Earth-Science Reviews* **88**, 145–166.

Briggs, J. C. 1995. *Global Biogeography*. Amsterdam, Elsevier.

Hammer, O. and Harper, D. A. T. 2006. *Paleontological data analysis*. Blackwell Publishing, Malden.

Hammer, O., Harper, D. A. T. and Ryan, P. D. 2001. *PAST*: paleontological statistics software package for education and data analysis. *Palaeontologia Electronica* **4**:1–9.

Hansen, C. B. 2007. Generalized least squares inference in panel and multilevel models with serial correlation and fixed effects. *Journal of Econometrics* **140**: 670–694.

Mosar, J., Lewis, G. and Torsvik, T.H. 2002. North Atlantic sea-floor spreading rates: implications for the Tertiary development of inversion structures of the Norwegian–Greenland Sea. *Journal of the Geological Society* **159**: 503-515.

Pinheiro, J., Bates, D., DebRoy, S., Sarkar, D., EISPACK, Heisterkamp, S., Van Willigen, B. and R Development Core Team. 2016. *nlme*: linear and nonlinear mixed effects models. *R package version* **3.1-126**: 1–336.

R Development Core Team. 2011. R*: a language and environment for statistical computing*. http://www.r-project.org.

Veizer, J., Godderis, Y. and François, L. M. 2000. Evidence for decoupling of atmospheric CO2 and global climate during the Phanerozoic eon. *Nature* **408**: 698–701.

Zachos, J., Pagani, M., Sloan, L., Thomas, E. and Billups, K. 2001. Trends, rhythms, and aberrations in global climate 65 Ma to present. *Science* **292**: 686–693.
